# Manual of Surgery

**Published:** 1904-06

**Authors:** 


**Manual of Surgery.** By Alexis Thomson, M.D., and Alexander Miles, M.D. Vol. I. Pp. 763. Edinburgh: Young J. Pentland. 1904.

The first volume of this manual is very excellent. The indications for treatment are very clearly laid down, and the book is very readable. It is also a pleasure to see some illustrations other than those which have a tendency to be handed down from one text-book to another. Amongst the more recent work we may notice a brief account of the value of the leucocyte count in surgery, the value of the X-ray treatment of rodent ulcer, the value of early movements and massage in the treatment of dislocations and some fractures, and a short mention of the Finsen light. A valuable note calls attention to the fallacies attending the diagnosis and position of fractures by skiagraphy. A mention is made of the very valuable suggestion of Jonathan Hutchinson, jun., to divide the glenoid ligament to aid in reducing the ordinary dislocation of the thumb. The treatment of burns is ably dealt with, the authors advising the use of picric acid in the early stages. Thiersch's grafting is well described, and a picture illustrating keloid on page 220 shows well what the result of the improper application of this method may be. These are only a few of the many points we could call attention to. If the high standard of this volume is maintained in the other (or others) we think it will be the most useful students' book on surgery that has recently appeared.

